# Validity and reliability of the Norwegian translation of the Achilles tendon Total Rupture Score

**DOI:** 10.1007/s00167-017-4689-1

**Published:** 2017-08-19

**Authors:** Ståle Bergman Myhrvold, Øystein Sandnes, Sigurd Erik Hoelsbrekken

**Affiliations:** 1 Department of Orthopedics, Akershus University Hospital, 1478 Lørenskog, Norway; 2 Department of Orthopedics, Ringerike Hospital, 3511 Hønefoss, Norway; 3 Department of Orthopedic and Rheumatic Surgery, Kongsvinger Hospital, P.O. Box 901, 2226 Kongsvinger, Norway

**Keywords:** ATRS, Achilles tendon rupture, Cross-cultural, Norwegian, Reliability, Validity

## Abstract

**Purpose:**

Patient reported outcome measures (PROMs) are increasingly being used in clinical research. The Achilles tendon Total Rupture Score (ATRS) is a PROM designed to assess outcomes in patients with ruptures of the Achilles tendon. The aim of this study was to develop a Norwegian adaption of the ATRS and evaluate its validity and reliability.

**Methods:**

The ATRS was translated into Norwegian in accordance with recommended guidelines. The study included 94 patients treated for Achilles tendon ruptures from January 2010 until February 2013, and the web-based questionnaires were administered twice. Reliability was assessed by Cronbach’s alpha, the intraclass correlation coefficient (ICC) and minimal detectable change (MDC). Construct validity was evaluated by calculating the Spearman’s correlation coefficient with the five subclasses of the Foot and Ankle Outcome Score (FAOS), the SF-36 subclass physical function and the SF-36 physical and mental summary scores.

**Results:**

Both internal consistency (Cronbach’s alpha = 0.96) and test–retest reliability (ICC = 0.90) were excellent, and the MDC was 2.12 at the group level and 16.98 at the individual level. Construct validity was supported by Spearman’s correlation coefficients above 0.7 with the SF-36 subclass physical function and the SF-36 physical summary score as well as the FAOS subclasses Sport/Rec and quality of life. There was no floor effect, and 6 patients (6.4%) achieved a maximum score of 100.

**Conclusions:**

The Norwegian adaption of the ATRS demonstrates acceptable validity and reliability for use in the Norwegian population to assess clinical outcomes in patients with Achilles tendon ruptures.

## Introduction

An acute rupture of the Achilles tendon represents a common injury [8], but the best choice of treatment remains controversial [6]. Recent studies emphasizing early mobilization have reported improved results after non-operative treatment [17], underlining the need to evaluate outcomes beyond occurrences of common complications such as re-ruptures, wound healing problems, infections and nerve injuries. Patient reported outcome measures (PROMs) are questionnaires answered by the patients themselves, and they are becoming increasingly popular when evaluating treatment results and patients satisfaction in clinical studies. The acute Achilles tendon Rupture Score (ATRS) is a PROM developed to assess outcomes in patients who have undergone treatment for acute Achilles tendon ruptures [13], and it has been validated in Swedish, English, Danish, Turkish, Persian, Dutch, Brazilian Portuguese and Italian [1, 4, 7, 9, 13, 15, 19, 21]. There are presently no PROMs in Norwegian validated for assessing outcomes after Achilles tendon injuries, and the purpose of this study was to translate and validate a Norwegian version of the ATRS. This will facilitate future research on the treatment of Achilles tendon ruptures in the Norwegian population.

## Materials and methods

**Fig. 1:**
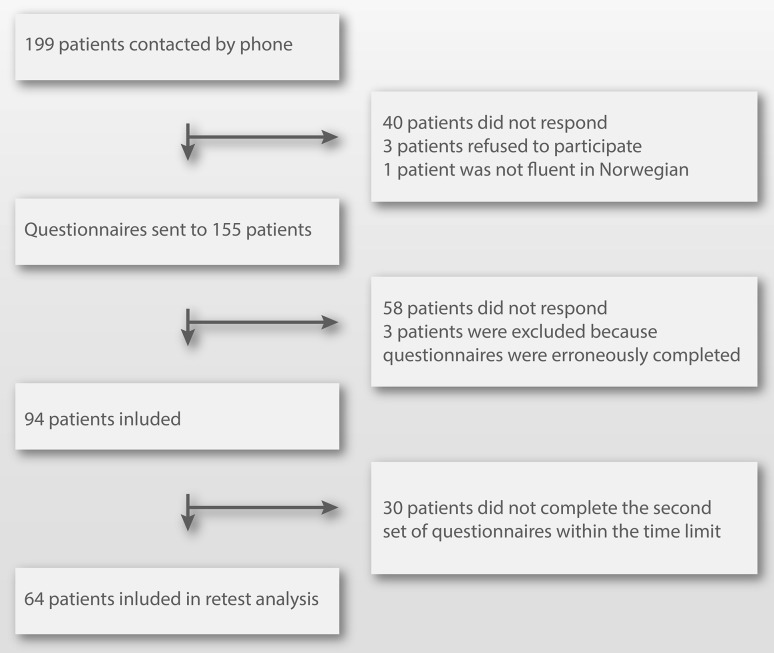
Inclusion flow chart

Patients were identified by searching the hospitals electronic admission record using the code for Achilles tendon rupture (S86.0) from the international classification of diseases, 10th revision (ICD-10). Patients aged 18–60 treated for acute ruptures of the Achilles tendon from 2010 until 2013 were considered eligible for inclusion, and a total of 155 patients gave consent for participation in the study (Fig. 1). There is no consensus regarding sample size calculations for the validation of PROMs, but we adhered to a recommended minimum of 50 patients [18]. The questionnaires ATRS, Foot and Ankle Outcome Score (FAOS) and the 36-Item Short Form Health Survey (SF-36) version 2 were completed online by logging on to a secured server. Patients who completed the questionnaires incorrectly were excluded from the study. Patients, who failed to complete the second set of questionnaires within four to eight weeks of completing the first set of questionnaires, were excluded from test–retest analysis. Patients reporting a change in their condition between completing the two sets of questionnaires were also excluded from test–retest analysis.

### Outcome measures and translations

The ATRS questionnaire contains ten questions, and each question is answered on an 11-point Likert scale ranging from 0 to 10. The total score is calculated by summing the individual Likert items. A score of 100 represents the absence of symptoms, whereas a score of 0 represents severe symptoms. The ATRS was translated into Norwegian according to recommended guidelines [2]. Three independent translators performed the translation from Swedish to Norwegian, and discrepancies were resolved by discussion. Two independent translators fluent in Norwegian and Swedish performed the back-translation into Swedish. The Norwegian language is very similar to Swedish, and the Norwegian version was reviewed and approved by the developer of the original Swedish ATRS.

The SF-36 is a self-assessment health status questionnaire composed of 36 questions sorted into eight multi-item scales. The SF-36 also provides two summarized measures represented by the physical component summary (PCS) and the mental component summary (MCS) [20]. The validity and reliability of the Norwegian translation of SF-36 have been found to be satisfactory [11, 12].

The FAOS questionnaire is a modification of the Knee injury and Osteoarthritis Outcome Score (KOOS). The only difference between KOOS and FAOS is the replacement of “knee” in KOOS with “foot/ankle” in FAOS [16]. FAOS consists of 42 questions divided into five subscales: pain, symptoms, function in daily living (ADL), function in sport and recreation (Sport/Rec) and foot- and ankle-related quality of life (QOL). Each subscale contains questions answered on a 5-point Likert scale ranging from 0 to 4. A normalized score (100 indicating no symptoms and 0 indicating severe symptoms) is calculated for each subscale. By replacing “knee” with “foot/ankle”, a Norwegian translation of FAOS from the Norwegian adaptation of KOOS in accordance with the original version of FAOS [16] was created.

### Reliability

Internal consistency indirectly evaluates whether different items in a questionnaire produce similar scores by measuring the correlation between the items. Poor internal consistency indicates the lack of correlation, which invalidates the creation of a summarized score. Cronbach’s alpha was used to evaluate internal consistency, and a Cronbach’s alpha greater than 0.7 was considered acceptable [3].

Test–retest reliability was defined as the ability of the questionnaires to measure the same outcome twice, and the ATRS, FAOS and SF-36 were completed at two different occasions with a washout period of four to eight weeks. The mean (SD) time between injury and completion of the questionnaires was 54.6 months (8.9) with a range of 36.1–72.7 months.

Test–retest reliability was calculated by the intraclass correlation coefficient (ICC) using a two-way random model, agreement and average measure (ICC 2.k). ICC was interpreted as follows: excellent (>0.75), fair to good (0.40–0.75) and poor (<0.40) [5].

Standard error of measurement (SEM) is the standard deviation of an observed test score, and there is a 95% probability that the persons “true” score is within ±2 × SEM of the observed score. Minimal detectable change (MDC) represents the smallest amount of change that can be detected beyond measurement error. SEM was calculated using the formula: standard deviation (SD) × √(1 − ICC). MDC at the individual level was calculated by 1.96 × √2 × SEM and at the group level by (1.96 × √2 × SEM)/√*n*.

### Construct validity

We evaluated criterion validity of the ATRS questionnaire by testing for correlations with the SF-36 component summaries PCS and MCS in addition to the subscale physical function (PF). We also calculated correlations between the ATRS and the five subscales of FAOS. Correlations were evaluated by use of the Spearman’s rank correlation coefficient as it is more robust to skewed data and outliers compared to Pearson correlation coefficient. Construct validity for ATRS was defined by hypothesizing a priori, correlation with SF-36 PF, SF-36 PCS, FAOS symptoms, FAOS Sport/Rec and FAOS QOL equal to or above 0.7, based on results from the Danish and Swedish validation studies [7, 13].

### Floor and ceiling effects

The presence of floor or ceiling effect was defined by more than 15% of the responders achieving the lowest or highest possible score, respectively [18].

### Ethics

The study was approved by the Regional Committee for Medical and Health Research Ethics of Norway (reference no. 2015/974).

### Statistical analysis

Categorical data were compared using the Chi-squared test. The Shapiro–Wilk test and inspection of histograms were used to test for normality, and the Levene’s test was used to assess equality of variances. Continuous variables showing normal distribution were presented with mean and SD and compared using the student *t* test or analysis of variance (ANOVA). Variables showing a non-normal distribution were presented with median and range. All analysis was performed in SPSS Statistics for Macintosh, Version 24.0 (Armonk, NY: IBM Corp).

## Results

### Demographics

Patient characteristics were comparable for non-responders and responders, and there were no significant differences between the groups (Table 1).

**Table 1:** Patient characteristics

	Non-responders *N* = 105	Responders *N* = 94	Retest ATRS *N* = 64
Male sex (%)	79 (75.2)	71 (75.5)	48 (75.0)
Mean age in years (SD)	45.3 (8.5)	46.7 (8.5)	46.8 (8.5)
Operative treatment (%)	92 (87.6)	89 (94.7)	61 (95.3)
Mean time between injury and completion of the questionnaires in months (SD)	54.3 (9.2)	54.9 (9.1)	54.5 (8.3)

### Translation

Norwegian and Swedish are closely related languages, and cultural differences are small. There was a single discrepancy in the forward translation of the ATRS regarding the translation of “Are you limited”. There are two different expressions that can be used interchangeably in Norwegian, and we chose the expression that most closely resembles the wording in the original Swedish version and that is also being used by the Norwegian translation of SF-36. There were no discrepancies in the back-translation of the ATRS.

### Reliability

The median (range) time between completion of the two set of questionnaires was 42 (28–56) days. Internal consistency for ATRS was 0.96, ICC was 0.90, and the SEM was 6.13. The MDC was 16.98 at the individual level and 2.12 at the group level.

### Validity

The ATRS demonstrated satisfactory correlations with the SF-36 component summary PCS and the SF-36 subscale PF as well as the FAOS subscales QOL and Sport/Rec, as defined by Spearman’s rank correlation coefficients above 0.7 (Table 2).

**Table 2:** Criterion validity of the ATRS assessed by Spearman’s rank correlation coefficient

	ATRS	*p* value
SF-36 PCS	0.72	<0.0001
SF-36 PF	0.71	<0.0001
SF-36 MCS	0.11	n.s.
FAOS Sport/Rec	0.81	<0.0001
FAOS QOL	0.76	<0.0001
FAOS ADL	0.66	<0.0001
FAOS pain	0.64	<0.0001
FAOS symptom	0.61	<0.0001

### Floor and ceiling effects

There was no floor effect observed for ATRS, and 6 patients (6.4%) achieved a score of 100. In contrast, the SF-36 subscale PF and several of the FAOS subscales displayed high ceiling effects (Table 3).

**Table 3:** Median values and ceiling effects

	Median (range)	Highest score *n* (%)
ATRS	87 (16.0–100)	6 (6.4)
SF-36 PCS	55.9 (30.2–62.8)	0 (0)
SF-36 MCS	55.5 (36.2–68.4)	0 (0)
SF-36 PF	87.5 (12.5–100)	45 (47.9)
FAOS Sport/Rec	90.0 (5.0–100)	32 (34.0)
FAOS QOL	87.5 (12.5–100)	26 (27.7)
FAOS ADL	98.5 (33.8–100)	44 (46.8)
FAOS pain	97.2 (33.3–100)	43 (45.8)
FAOS symptom	89.3 (35.7–100)	20 (21.3)

## Discussion

The main finding of the study was acceptable validity and reliability demonstrated by the Norwegian adaptation of the ATRS. The Norwegian translation of the ATRS can therefore be used in the Norwegian population to assess clinical results in patients sustaining ruptures of the Achilles tendon.

The observed internal consistency was comparable to previous reports from other cross-cultural adaptations of the ATRS ranging from 0.89 to 0.97 [1, 4, 7, 9, 13, 15, 19]. The SEM was also in agreement with previously reported results ranging from 3.2 to 10.91 [1, 4, 7, 9, 15]. The MDC of 16.98 at the individual level was considerably lower than 30.24 reported by the Dutch validation study [15], but similar to 18.5 observed in the Danish study [7]. The MDC of 2.12 at the group level was lower compared to the Dutch and English adaptations with 3.49 and 6.75, respectively [4, 15]. To our knowledge, MDC has only been reported by the Danish, Dutch and English validation studies. Based on the MDC values, the Norwegian translation of the ATRS is well suited to compare groups of patients as a difference above 2.12 points reflects a real change, but the individual MDC of 16.98 restricts its usefulness in the follow-up of individual patients. The ICC was similar to 0.908 attained in the Danish validation and well within what has previously been reported (0.852–0.986) [1, 7, 13, 15, 19].

There are no PROMs validated in Norwegian for assessing clinical outcomes after Achilles tendon ruptures, and the Norwegian adaptation of the ATRS could not be correlated with an outcome measure specific for Achilles tendon ruptures. We therefore correlated the ATRS with SF-36 domains equivalent to the Danish adaptation [7]. We also tested for correlations with all five FAOS subscales similar to the Swedish validation study [13]. The correlation data attained in the present study were similar to the results from the Swedish and Danish validations [7, 13]. This was in accordance with expectations since Danish, Norwegian and Swedish are closely related languages, and cultural differences between the countries are small. The correlation coefficients between the Norwegian ATRS and the SF-36 PCS and SF-36 PF were all above 0.7, as hypothesized. The correlation coefficients were also above 0.7 with the FAOS subscales QOL and Sport/Rec. Although the correlation coefficient was below 0.7 with the FAOS subscale symptoms, four out of five a priori hypothesized correlations were confirmed by the study, which is considered acceptable [18].

None of the patients attained an ATRS score of 0, and thus, no floor effect was present. A ceiling effect was observed in 6.4% of the patients, but this was below the pre-defined threshold of 15%. In contrast, the SF-36 subscale PF and all the FAOS subscales except for symptoms demonstrated ceiling effects well above 15%. Unfortunately, neither the Dutch, Swedish or Turkish validation study reported ceiling data for the FAOS subscales [9, 13, 15], but such high ceiling effects do question the suitability of the Norwegian adaptation of FAOS to assess clinical results in patients sustaining Achilles tendon ruptures. We did not evaluate sensitivity to changes over time for the Norwegian ATRS, but responsiveness has been evaluated for the original version [4, 10].

The time period between the two test occasions was longer in the present study compared to previous ATRS adaptations, which may potentially have influenced reliability testing. However, the mean time from injury to answering the first questionnaire was 54.9 months in the present cohort, and only minor clinical improvements can be expected more than one year after injury [14]. This indicates that the cohort was fairly homogenous with respect to state of rehabilitation, and results from test–retest analysis were similar to what has previously been reported.

Only 94 out of 199 patients (47%) eligible for inclusion completed the first set of questionnaires. The low response rate may have introduced selection bias, and we are unable to account for the non-responders apart from characteristics provided in Table 1. It is therefore difficult to ascertain that the included group of patients is representative of the general population. Selection bias may also have been introduced in test–retest analysis as only 64 out of 94 patients (68%) completed the second set of questionnaires.

The results from this study are similar to previous adaptations of the ATRS, and both the validity and reliability of the Norwegian ATRS were acceptable. This allows for follow-up and comparison of different treatment options using a PROM adapted to the Norwegian language that is designed to evaluate results after Achilles tendon ruptures. It will also facilitate future research on the treatment of Achilles tendon ruptures in the Norwegian population.

## Conclusion

The Norwegian adaptation of the ATRS demonstrates acceptable validity and reliability for use in the Norwegian population to assess the clinical outcome in patients with Achilles tendon ruptures.

## Abbreviations

PROM: Patient reported outcome measure
ATRS: Achilles tendon Total Rupture Score
ICC: Intraclass correlation coefficient
FAOS: Foot and Ankle Outcome Score
SF-36: Short Form-36 version 2
KOOS: Knee injury and Osteoarthritis Outcome Score
PCS: Physical component summary of the SF-36
MCS: Mental component summary of the SF-36
PF: Physical Function Score of the SF-36
Sport/Rec: Function of sport and recreation subclass in FAOS
ADL: Function in daily living
QOL: Foot- and ankle-related quality of life subclass in FAOS
SEM: Standard error of measurement
SD: Standard deviation
MDC: Minimal detectable change
